# Effect of Shear Angle and Printing Orientation on Shear Constitutive Response of Additively Manufactured Acrylonitrile Butadiene Styrene

**DOI:** 10.3390/polym14122484

**Published:** 2022-06-18

**Authors:** Joshua Letizia, Vijaya Chalivendra, Dapeng Li

**Affiliations:** 1 Department of Mechanical Engineering, University of Massachusetts, Dartmouth, MA 02747, USA; jletizia@umassd.edu; 2 Department of Bioengineering, University of Massachusetts, Dartmouth, MA 02747, USA; dli@umassd.edu

**Keywords:** shear constitutive behavior, shear angle, printing orientation, modified flat-hat shaped specimen, acrylonitrile butadiene styrene (ABS)

## Abstract

An experimental investigation was performed to understand the quasi-static shear response of additively manufactured (AM) acrylonitrile butadiene styrene (ABS) via fusion deposition modeling (FDM). A modified flat hat-shaped (FHS) specimen configuration was used for shear testing. The main aim of this study was to investigate the effect of four different shear angles (0°, 5.44°, 13.39°, and 20.83°) and three printing orientations (vertical build, 0°/90°, and 45°/−45°) on the shear constitutive response and shear performance of FDM-printed ABS. Scanning electron microscopy images of the failure surface were used to explain the shear response of the material. The flow shear stress of the shear stress-strain response for vertically printed specimens demonstrated a monotonic increase up to a peak shear stress and then decrease at the end of the shear zone, while for 0°/90° specimens, an increasing trend until the peak value at the end of the shear zone was observed. With increasing shear angles, all specimens printed with three printing orientations exhibited increasing shear zone size and shear strength, and the 0°/90° specimens exhibited the highest shear strength for all four shear angles. However, the specimens of the 45°/−45° orientation demonstrated the highest increase in shear strength by about 60% and in the shear strain at the end of shear zone by about 175% as the shear angle was increased from 0° to 20.83°.

## 1. Introduction

Additive manufacturing (AM), generally known as 3D printing, has advanced to a great extent in the last decade. It has many advantages, particularly in its capability of prototyping or fabricating customized products of complex structures and shapes without having to use any additional tools or processing [1]. Out of all polymer-based AM techniques, fusion deposition modeling (FDM) has been widely used as functional parts in manufacturing and assembly models [2,3,4,5,6,7,8]. Among various thermoplastic polymer-based filament materials for FDM, acrylonitrile butadiene styrene (ABS) has been widely studied and used in developing functional prototypes due its high strength, toughness, light weight, and resilience [9]. Many studies on tensile, compressive, flexural, and fracture characterization of 3D-printed ABS with various printing parameters have been reported in literature. Rodriguez et al. [10] studied the mechanical behavior of FDM printed ABS under tensile loading, where Young’s modulus decreased by 11% to 37%, and the tensile strength reduced by 22% to 57% relative to an ABS monofilament. Ahn et al. [11] investigated the effect of raster orientation, air gap, bead width, and model temperature on tensile and compressive anisotropic mechanical properties of ABS specimens printed in different directions. For an overlap of 76 µm between the roads, the tensile strength ranged between 65% and 72% to those of injection molded ABS. Abbot et al. [12] studied the impact of nozzle temperature, print speed, and layer height on the tensile strength of ABS and investigated the bond strength between the fused filaments by analyzing the tensile strength measurements. It was reported that print speed had a large impact on the bond strength, and higher speeds resulted in lower strength. Samykano et al. [13] studied the effect of three printing parameters, including layer height, raster angle, and infill density, on the mechanical properties of FDM-printed ABS. The authors identified optimum parameters of 80% infill percentage, 0.5 mm layer thickness, and 65° raster angle, which improved all tensile properties. Lee and Huang [14] compared the fatigue response of FDM-printed ABS to that of bulk ABS using the UNI EN ISO 527-1 standard. The FDM-based ABS experienced fatigue failure in the order of thousands of cycles at 40% of ultimate tensile strength (UTS), while the bulk ABS experienced failure at 60% of its UTS at the identical cycle limit. He and Khan [15] studied bending fatigue behavior for FDM-printed ABS beams of various building orientations, layer thicknesses, and nozzle sizes and at various thermo-mechanical loading conditions. An optimum parameter combination of 0° building orientation, 0.15 mm layer thickness, and 0.8 mm filament width demonstrated the longest fatigue life before the failure at each temperature condition. Abdurrahman and Fitri [16] conducted fatigue strength analyses of FDM-printed ABS shafts under rotating bending loads and found that the S-N curves of ABS showed a maximum average number of cycles of 143,702 at a stress of 26.87 MPa and minimum average number of cycles of 145 at a higher stress of 35.71 MPa.

Aside from virgin ABS, 3D-printed ABS composites reinforced by different fillers have also been studied and reported. Sezer and Eren [17] prepared 7 wt.% multi-wall carbon nanotubes (MWCNTs) reinforced ABS, which exhibited enhanced tensile strength and electrical conductivity by about three-fold and seven-fold, respectively. Akhoundi and Behravesh [18] determined the effect of filling pattern (concentric, rectilinear, hilbert curve, and honeycomb) and infill percentage on the tensile and flexure properties of FDM-printed polymers. Their results indicated that a concentric pattern provided the highest tensile and flexural tensile properties for all filling percentages. Weng et al. [19] studied the effect of addition of Montmorillonite (OMMT) nanoplates to ABS on the thermo-mechanical properties of the particulate composite materials. These composites demonstrated superior tensile modulus, flexural modulus, flexural strength, and dynamic mechanical storage modulus over those of their injection molded counterparts. Camargo et al. [20] characterized the mechanical and electrical behaviors of graphene-reinforced ABS, which were FDM-printed with various infill and layer thickness parameters. The addition of graphene turned out to decrease both the tensile yield strength and flexural strength by about 50%. Hamaz et al. [21] fabricated copper ferrite (CuFe_2_O_4_) nanoparticle-reinforced ABS composites also via FDM, showing 135% and 14% improvement in tensile strength and stiffness, respectively, for the ABS-CuFe_2_O_4_ composite with 14 wt.% CuFe_2_O_4_, over virgin ABS.

In addition to tensile and flexural, fracture properties of FDM-printed ABS were also reported in the recent past. McLouth et al. [22] studied the impact of three orthogonal orientations and the raster patterns +45/−45° and 0/90° of FDM-printed ABS on fracture toughness and found a 54% fracture toughness increase in the samples having filament orientation perpendicular to the crack plane compared to those of filaments parallel to crack plane. Li et al. [23] conducted both experimental and numerical fracture studies to investigate the effect of printing orientation (vertical build, 0°/90°, and 45°/−45°) on fracture toughness of FEM-printed ABS. They discovered that fracture toughness was highly dependent on printing orientations and a crack kinking phenomenon was observed in 45°/−45° print orientation. Additionally, numerical parametric studies demonstrated that the inter-filament bonding strength could be adjusted to generate different crack paths for the dissipation of maximum fracture energy. Hart et al. [24] was able to increase the fracture toughness of FDM-printed ABS by 2700% through annealing, presumably due to thermally driven void migration and aggregation. Rabbi et al. [25] employed novel surface topology patterns to improve the dynamic fracture initiation toughness of FDM-printed ABS of various printing orientations. The presence of surface patterns resulted in a 58% increase in fracture toughness compared to those without patterns. Besides, further improvement in fracture toughness was achieved by increasing the size of the circular pattern, as well as by changing the pattern shape to square. More recently, Ameri et al. [26] predicted fracture loads of FDM-printed ABS specimens under mixed-mode fracture loading conditions. In this study, the authors compared the fracture loads measured through experiments with those of Maximum Tangential Stress (MTS) and Generalized Maximum Tangential Stress (GMTS) criteria. The predicted fracture loads using the GMTS criterion were closely matched with those from experiments. Nabavi-Kivi et al. [27] investigated the influence of printing speed and mode-mixity on the fracture response of FDM-printed ABS. The highest mixed-mode fracture toughness was observed from the specimens with a printing speed of 50 mm/s.

Despite many studies on tensile, compression, flexure, and fracture characterizations of FDM-printed ABS, no attempts have been made in understanding its shear constitutive behaviors to date, due to the challenge involved in identifying a specimen configuration suitable for testing for shear deformation. In this study, a modified flat hat-shaped (FHS) specimen configuration was used to determine the shear constitutive response of FDM-printed ABS; a parametric study was conducted to investigate the effect of printing orientation and shear angle on the shear strength. The major novelty of this study was to identify a suitable specimen configuration to understand the shear behavior in FDM-printed polymers and investigate an optimum shear angle and raster orientation for superior shear performance. Scanning electron microscopy (SEM) imaging was employed to gain insights into the failure mechanisms.

## 2. Experimental Details

### 2.1. Material and Specimen Design

**Figure 1:**
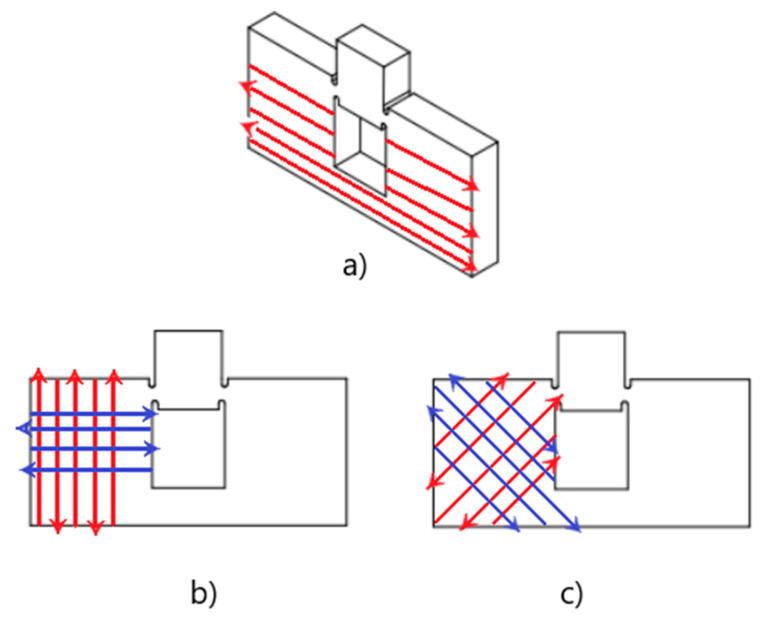
Sketch of specimen configurations with their respective raster orientation sequences (**a**) Vertical; (**b**) 0°/90°, with 0° oriented in blue and 90° oriented in red; (**c**) 45°/−45° with 45° oriented in red and −45° in blue.

A modified flat hat-shaped (FHS) specimen, as shown in Figure 1, was fabricated utilizing a uPrint 3D printer (Stratasys, Eden Prairie, MN, USA). This modified FHS specimen configuration was adopted from cracked FHS, which was used for determining the dynamic fracture toughness of metals by Xu et al. [28]. In this study ABS-P430 (Stratasys, Eden Prairie, MN, USA) was considered because it is strong, rigid, resistant to abrasion, and impact-resistant. It is widely used in functional prototyping, jigs, fixtures, pipe fittings, auto parts, and electronic housings. In general, ABS is a terpolymer consisting of three monomers: acrylonitrile; butadiene; and styrene. The amount of each monomer in the ABS can range from 15% to 35% acrylonitrile, 5% to 30% butadiene, and 40% to 60% styrene. Although the ABS-P430 filament is commercially available, it is a proprietary mixture; hence, the exact percentage of each monomer is not publicly disclosed. The melt flow index (MFI) of ABS-P430 is 2.4 g/10 min. The tensile strength of the ABS-P430 is 36 MPa, the tensile modulus is 2.4 GPa, and the tensile elongation at break is 4%.

Beforehand, the specimens were designed and configured for fabrication utilizing a computer-aided design software, SolidWorks. Once completed, the file was exported in the form of a stereolithography (STL) file, which was then sent to the uPrint. The Stratasys’ 3D printer utilized a printing software called CatalystEx to interpret the STL file and eventually fabricate the test sample. It employed a process of discretization, which essentially slices the specimen and builds a blueprint of each layer corresponding to the printer’s extruder. This extruder is guided along a path predetermined by the software of the printer through the generation of a G code. This G code directs the printer to follow the predetermined extrusion path, which is also known as printing or raster orientation. All specimens of three different orientations were printed with the same extrusion parameters, which are listed with details in Table 1 to ensure consistent printing processing conditions. These parameters were provided by Stratasys to generate the optimum quality of printed parts of ABS. Figure 1 provides the schematic of three different printing orientations (vertical, 45°/−45°, 0°/90°) considered in this study. For specimen material, Acrylonitrile butadiene styrene (ABS) was considered, as it is widely used in industrial applications.

**Figure 2:**
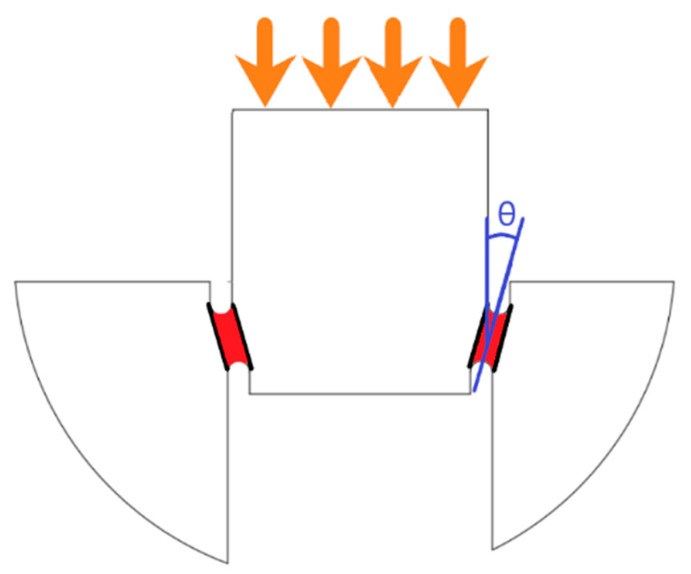
Schematic of specimen showing the shear notch zones (red) with a shear angle (θ).

**Figure 3:**
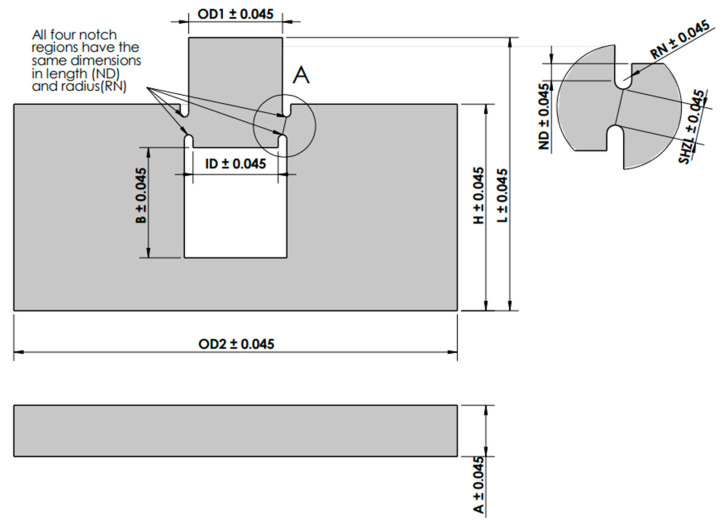
Flat Hat-Shaped Specimen illustrated with given dimensions and detailed variables given in Table 2.

In this study, in addition to three raster orientations, four shear angle orientations (0°, 5.44°, 13.39°, and 20.83°) were incorporated into the specimen to analyze the effect of the shear angle on the shear deformation and shear performance of the ABS. An illustration of shear angle in the shear zone of the test specimen is shown in Figure 2. A detailed illustration of the shear test specimens used in this study with all dimensions is given in Figure 3. The geometric dimensions used for various shear angles of test specimens are given in Table 2. These shear angles were chosen based on the distance between OD1 and ID of the hat shown in Figure 3. For similar values of OD1 and ID, the shear angle was 0°; for a difference of 0.4mm, the shear angle was 5.44°; for a difference of 1 mm, the shear angle was 13.39°; and for a difference of 1.6 mm, the shear angle was 20.83°.

### 2.2. Shear Characterization

**Figure 4:**
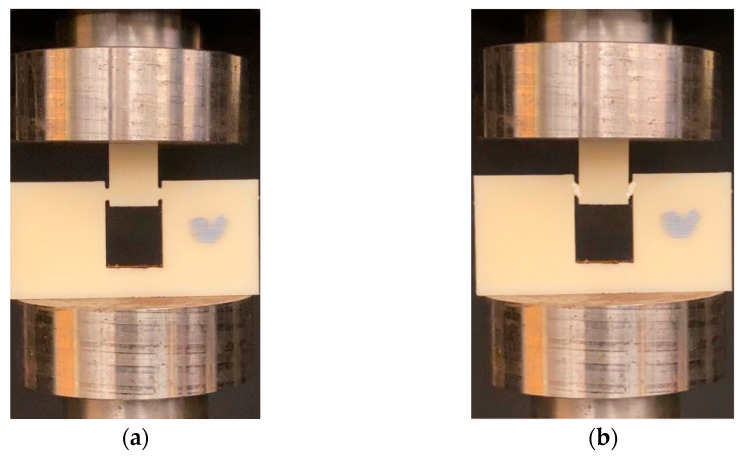
Vertical sample of Flat Hat-Shaped specimen at (**a**) no load condition and the (**b**) shear-deformed configuration.

Experiments were performed on the shear test specimens using an INSTRON 5569 (Instron, Norwood, MA, USA) in displacement mode. As shown in Figure 4, the specimens were placed between two compression platens, where the top platen was moved at a displacement rate of 1.27 mm/min. The experiments were done at room temperature, where load-point displacements and load values were recorded for each test specimen. The compression of the top hat induced pure shear deformation between the notches in the shear zone (as shown in Figure 2). Using the MicroCapture^®^ Pro digital microscope (Instron, Norwood, MA, USA), the shear deformation was captured for the entire experiment duration. A minimum of six identical experiments were performed for each configuration to have statistical significance. Using load-point displacement and applied compressive load, the shear strain and shear stress were calculated using the following equations:

(1) $$\gamma =\frac{tan^{-1}\left(d\times cos\left(\theta \right)\right)}{shzl}$$

(2) $$\tau =\frac{F\times \left(cos\left(\theta \right)\right)}{A_{S}}$$

where *γ* is the shear strain, *d* represents load-point displacement, *θ* represents the shear angle, and *shzl* represents the length of the shear zone between the two notches. *τ* represents the shear stress, while *F* is the applied compressive force, and *A_S_* represents the shear area of the test specimen.

## 3. Results and Discussion

**Figure 5:**
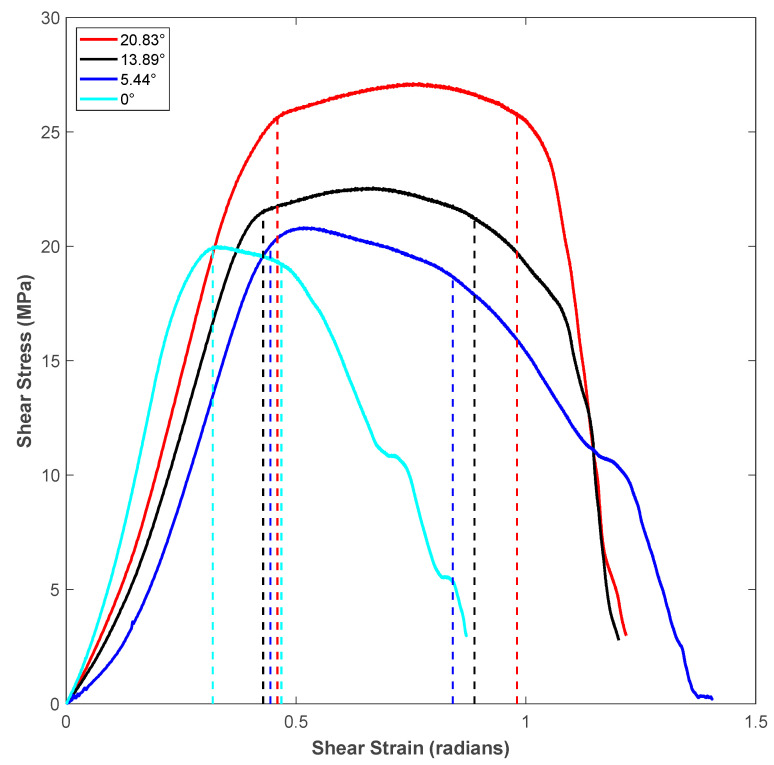
Shear stress-strain response for specimens of different shear angles for Vertical raster orientation.

**Figure 6:**
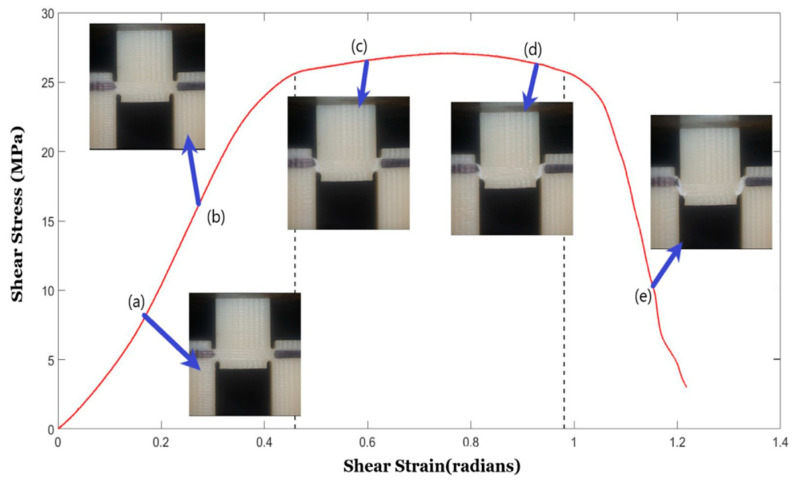
Example of the Shear zone in a vertical raster-oriented print provided with real time photos from video recording taken at each section in the graph. Sections are denoted by the corresponding letters of arrows; (**a**,**b**) represent the specimen undergoing small visually unnoticeable shear deformation, (**c**,**d**) represents the shear zone in which the specimen begins to deform significantly, and (**e**) represents the sliding of the sheared piece.

Figure 5 represents the shear stress-strain response for specimens of different shear angles for vertical raster orientation. Considering statistical significance, a minimum of five specimens were tested for each shear angle of a given raster orientation. A representative curve for each shear angle was selected for plotting in the figure. The region between the dotted vertical lines drawn for each stress-strain curve of different shear angles represents the shear zone. The first dotted vertical line denotes the start of the noticeable shear deformation, and the second dotted vertical line denotes the end of shearing and the start of sliding of the sheared piece. The stress-strain response before the first dotted vertical line was nearly linear, and during this stage, shear stress was built up within the shear zone of the test specimen to generate noticeable shear deformation, which was captured using the imaging system. Figure 6 provides a sample of sequential specimen images of the video recorded in relation to the shear stress-strain response. The response of the sample was broken up and characterized by the following three zones: sample with small visually unnoticeable shear deformation, sample under significant shearing deformation (shear zone), and sample under sliding (sliding zone). During the first zone, the specimen underwent nearly linear elastic deformation before it became nonlinear at the end of this zone. In zone-2, the sample experienced a significant amount of shear deformation nearly at constant flow stress. The visual change of shear zone was noticeable due to the color variation of the specimen in the two notch regions undergoing shear deformation. This color variation is associated with plastically deformed polymer chains of the printed filaments. The final stage (zone-3) was characterized by the rapid descent in loading and the sample beginning to slide. During the sliding, the test specimen failed to withhold the applied load, leading to the separation of hat portion from rest of the specimen.

It can be noticed in the shear zone of Figure 5 that the specimen experienced maximum shear stress (shear strength) at a later part of the shear zone as the shear angle of the specimen increased. Moreover, both the shear flow stress within the shear zone and the length of the shear zone increased as the shear angle of the shear test specimen increased. The measured values of shear zone length (in terms of units of shear strain, which is in radians) for different shear angles is shown in Table 3. The shear zone increased by about 3.5-times when the shear angle increased from 0° to 20.83° for specimens of vertical roster orientation. This could be explained by the fact that the incremental shear angle gave way for more filament in the specimen’s shear region, which led to an increased length of the shear zone of the flat hat specimen, as listed in Table 2. During the sliding portion of the stress-strain diagram of each shear angle, the shear stress dropped as the material in the shear zone was no longer connected to the supports of the specimen.

**Figure 7:**
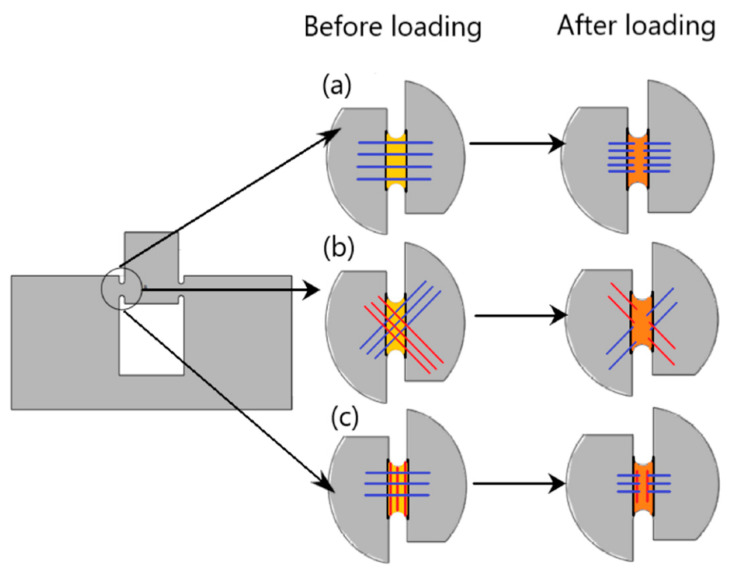
Schematic representation of filaments before and after loading for each raster orientation; (**a**) Vertical, (**b**) +45/−45°, and (**c**) 0/90°.

**Figure 8:**
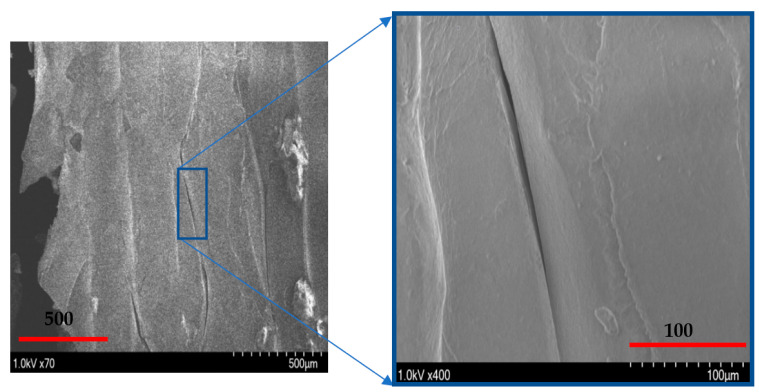
Scanning electron microscope image of the shear zone in the Vertical orientation with a 0° shear angle.

A schematic of the failure of extruded filaments for test specimens of each raster orientation is shown in Figure 7. For vertical raster orientation, as shown in Figure 7a, the extruded filaments of horizontal direction got sheared off under shear deformation. In Figure 7b, shown for 45°/−45° raster orientation, the filaments of either 45° or −45° were sheared off. Finally, for specimens of 0°/90° raster orientation, shown in Figure 7c, the filaments of 0° were sheared off, whereas those of 90° remain intact. Figure 8 shows the image of scanning electron microscopy (SEM) of vertically printed test specimens. It is clearly evident from the failure surface that the filaments that were oriented horizontally in the vertical built were sheared with a large deformation. During this process of shearing, the gaps that typically exist between the extruded filaments while printing were also closed. Moreover, the failure surface of sheared filaments showed a smooth surface due to less resistance offered by the filaments compared to the other two raster orientations, as the shear flow stress gradually reached a maximum value and decreased steadily until the end of the shear zone, as shown in Figure 5.

**Figure 9:**
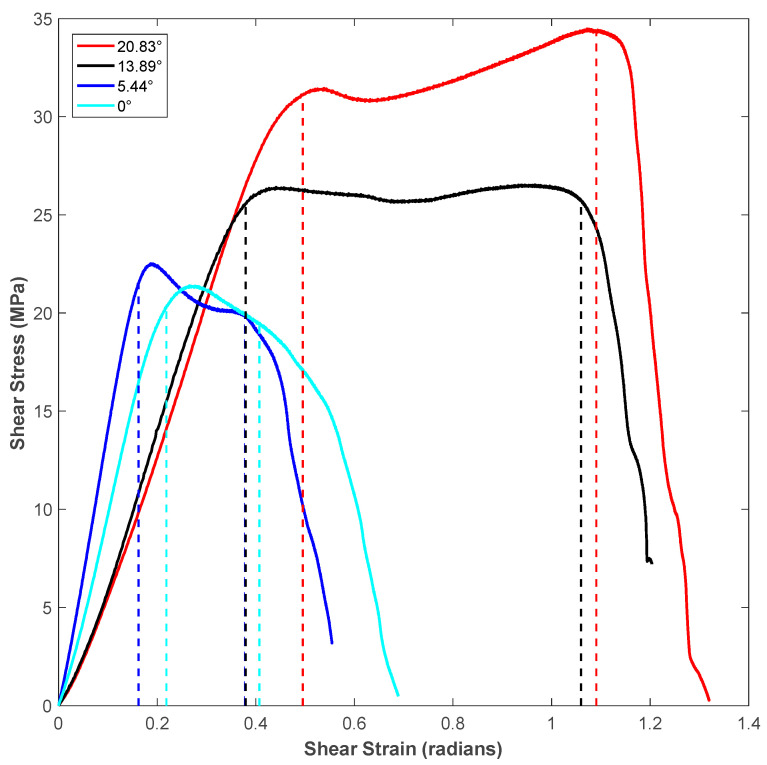
Shear stress-strain response for the +45°/−45° raster orientation with all four notch angle configurations.

**Figure 10:**
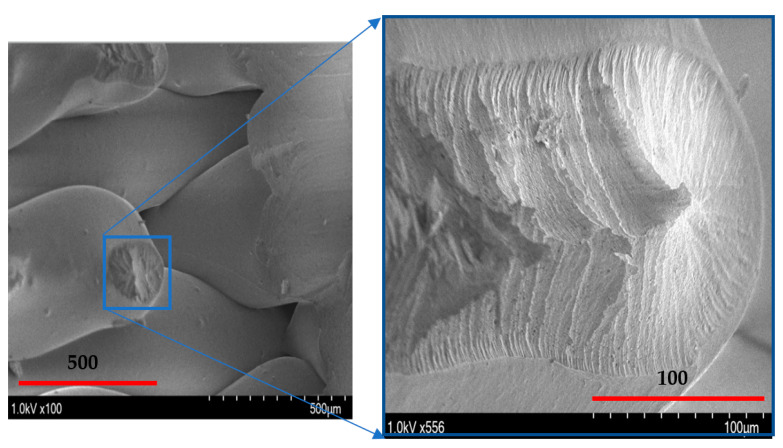
Scanning Electron Microscope (SEM) images of a 45/45° raster orientation, with a 5.44° notch angle specimen. (**left**) depicts the zoomed out and (**right**) depicts the zoomed in localized area of shear deformation.

Figure 9 shows the shear stress-strain response of the printing raster orientations of +45°/−45°. The shear stress-strain responses demonstrated a similar trend to those of vertical raster orientation as the shear angle increased. However, unlike vertical orientation, the shear stress-strain curve demonstrated an initial increase and a slight drop before a further increase during the shear zone. This variation was attributed to the +45°/−45° pattern of filament orientation, where the extruded filaments offered slight compliance in the shear flow stress before increasing the slope, especially for the higher shear angles of 13.39° and 20.83°. As provided in Table 3, like the vertical orientation, the shear zone length also increased with the shear angle for the +45°/−45° orientation. In general, the shear zone size was slightly higher for the +45°/−45° orientation compared to vertical orientation. During the shear deformation in the +45°/−45° orientation, the extruded filaments of both +45° and −45° directions underwent shear failure, as shown in the schematic of Figure 7b. The SEM images of the shear failure surface of the test specimen for +45°/−45° orientation are shown in Figure 10. The failure surface indicated the highly rough surface with several ridge markings compared to the vertical orientation. This could be related to the shear flow stress variation during the shear zone, as shown in the Figure 9, where the increase in the slope of the flow stress was associated with energy required to deform and shear off the extruded filaments. During this process, the filaments undergo a large amount of localized deformation, which results a rough failure surface, as shown in Figure 10. In the literature, the fracture surface of FDM-printed ABS of +45°/−45° orientation under mixed-mode (a combination of opening and shear loading) fracture conditions demonstrated fewer ridge markings on the broken filaments [27]. However, several ridge markings were noticed in this work on the failure surface of the specimens subjected to shear loading.

**Figure 11:**
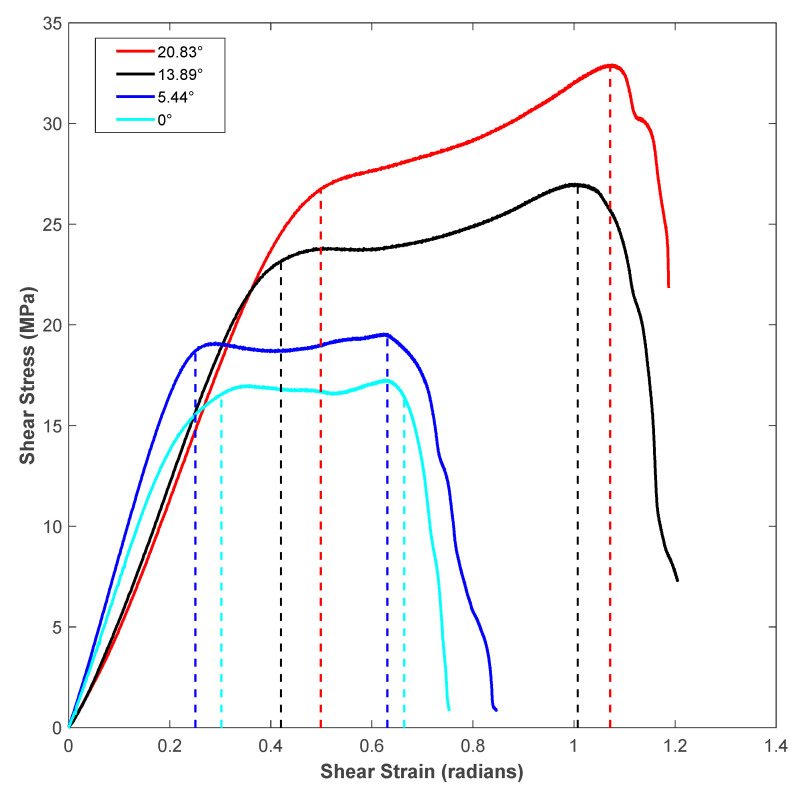
Shear stress-strain response of the 0/90° raster orientation with all four notch angle configurations.

**Figure 12:**
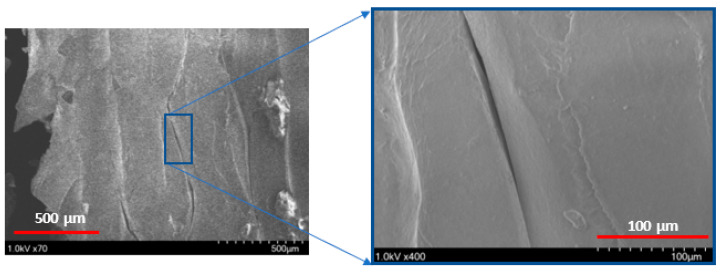
Scanning electron microscope snapshot of shear zone in the 0°/90° orientation with a 20.83° shear angle (**left**) zoomed; the 0° filaments are in orange. The 90° filaments are in red. Close up of the shear zone (**right**).

Figure 11 provides the shear stress-strain response of all four shear angles for specimens of the 0°/90° raster orientation. Like the other two orientations, the shear flow stress and length of the shear zone increased as the shear angle increased. Most importantly, the size of the shear zone for 0/90° at a shear angle of 0° was much higher than the other two raster orientations, as given in Table 3. This was due to a higher resistance for shearing of the extruded filaments of 0° in the presence of 90° extruded filaments. If this value was compared with vertical raster orientation, where all the extruded filaments were along the horizontal direction, they got sheared with less than half of the shear zone length than that of 0/90° raster orientation. Furthermore, this reason could be reinforced with the nature of the shear stress-strain diagram of the 0/90° raster orientation, where the shear stress did not drop off after reaching the maximum value for the shear angle of 0°. However, in the case of the other two orientations, the shear stress value dropped as soon as it reached the maximum value. One other major difference was that the specimens of the 0/90° raster orientation showed peak shear stress at the end of the shear zone compared to all other raster orientations. This could be explained by the fact that for the 0/90° raster orientation, the extruded filaments in the 0° direction sheared off, while they were sandwiched between the two extruded filaments of the 90° direction. As shown in SEM images of Figure 12, the extruded filaments along the 0° underwent severe localized deformation. While these filaments experienced large deformation, the filaments in the 90° offered constraint. This constraint was demonstrated by the increase in shear stress as the shear strain increased, until the specimen reached the maximum shear stress value, as shown in Figure 11. In comparison, the failure surface of FDM-printed ABS of a 0/90° orientation under tensile loading demonstrated brittle failure of the filaments along 0°. Moreover, the pores between the 0° and 90° filaments were intact, even after failure of the specimen [29].

**Figure 13:**
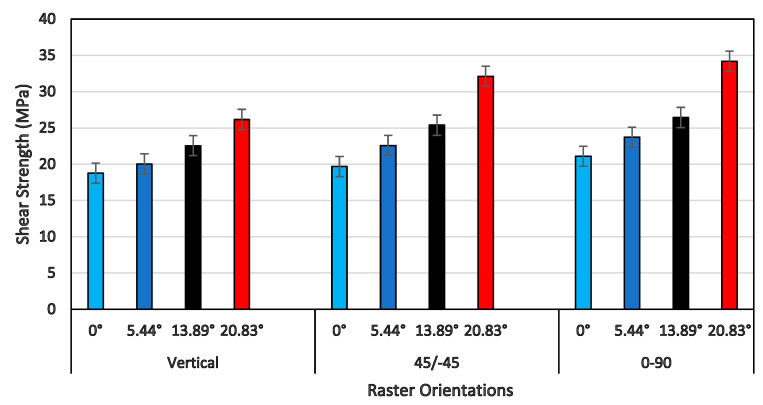
Shear strength with their respective raster orientations and shear angles.

A comprehensive plot of the shear strength of all three raster orientations and four shear angles is shown in Figure 13. In general, the shear strength increased as the shear angle increased for all three raster orientations. However, the shear strength increased only by about 50% for vertical orientation, by about 63% for the +45°/−45° orientation, and by about 55% for the 0°/90° orientation, as the shear angle increased from 0° to 20.83°. Although the percentage increase of shear strength for 0°/90° was less than that of the +45°/−45° orientation, the values of the shear strength for all four shear angles of 0°/90° were slightly higher than those of all of the other two orientations. This was due to the presence of a constraint provided by the extruded filaments of the 90° direction, as the filaments of 0° underwent large-scale shear deformation and failure. This was clearly evident from the shear stress-strain response that the shear flow stress increased as the shear strain increased, and the maximum shear stress happened at the end of the shear zone (shown in Figure 11) against other two raster orientations, except for a specimen of a shear angle of 20.83° for the +45°/−45° raster orientation. When the shear strength values shown in Figure 13 were compared with tensile strength values of the 0°/90° and +45°/−45° orientations, the shear strength for the shear angle of 0° was about 65% less than that of the tensile strength for both raster orientations [27,29].

**Figure 14:**
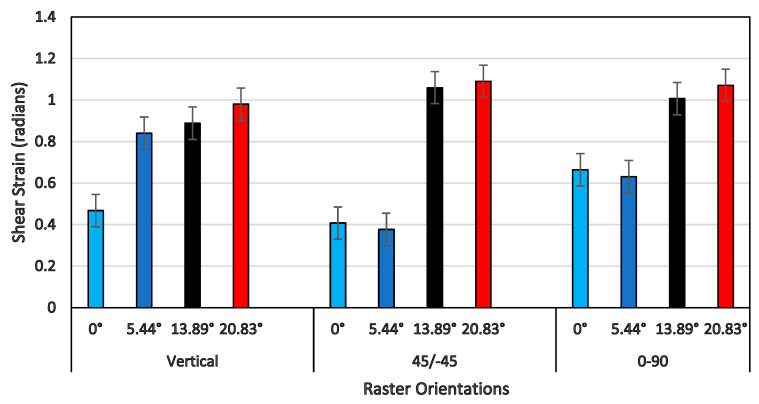
Shearing strain of the shear zone endpoints (in radians) with their respective raster orientations and shear angles.

**Figure 15:**
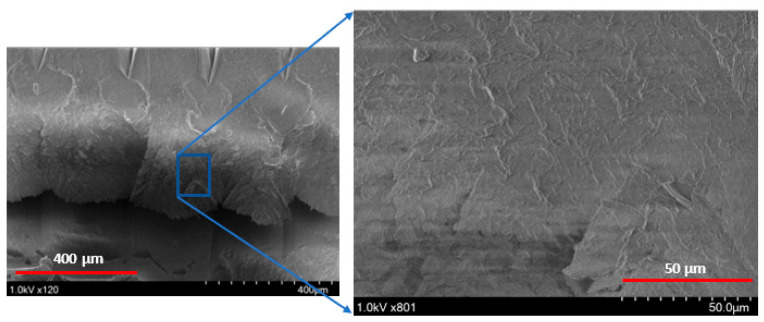
Scanning electron microscope snapshot of the shear zone in the +45°/−45° orientation with a 13.39° shear angle, (**left**) zoomed out. Close up of the shear zone (**right**).

Figure 14 provides the shear strain at the end of the shear zone for all shear angles and raster orientations. For a vertical raster orientation, the shear strain value showed a sharper increase by 75% when the shear angle increased from 0° to 5.44°. Beyond the shear angle of 5.44°, the increase in shear strain was steady. This shear angle can be called a critical shear angle for vertical orientation. For the other two raster orientations (+45°/−45° and 0°/90°), the critical shear angle associated with the sharper rise in the shear strain value was 13.89°. Out of these two orientations, the highest increase of a 200% increase in the shear strain value was noticed for the +45°/−45° orientation, when the shear angle changed from 5.44° to 13.89°. The failure surface imaged from SEM of a shear angle of 13.89° indicates that the specimen underwent severe localized shear deformation, as shown in Figure 15. Conversely, at the shear angle of 5.44°, the filaments experienced failure with a rough surface (shown in Figure 10), indicating relatively brittle failure compared to that of 13.89°. Although ultimate tensile strain values have been reported in the literature for the +45°/−45° orientation [29], they could not be compared with the maximum shear strain values at the end of shear zone due to the nature of how they are determined.

## 4. Conclusions

A detailed experimental study was conducted to investigate the shear behavior of an FDM-printed ABS polymer under quasi-static shear loading conditions. The effect of raster orientations and the shear angles on the shear stress-strain response, shear zone, and the shear strength was investigated. The following were the major outcomes of this study:

As the shear angle increased, the shear zone size and shear strength increased for all three raster orientations.

The flow shear stress in the vertical raster orientation during the shear zone demonstrated a monotonic increase up to a peak shear stress and then a decrease at the end of the shear zone. However, the flow shear stress showed an increasing trend until the peak value at the end of the shear zone for the 0°/90° raster orientation.

The shear strengths of all four shear angles of the 0°/90° raster orientation were slightly higher than those of the other two orientations due to the constraint provided by the extruded filaments in the 90° direction, while the extruded filaments of the 0° direction underwent large-scale plastic deformation, as noticed in SEM images.

The shear strain value at the end of the shear zone demonstrated a sharp increase at a certain critical shear angle for all three orientations. The +45°/−45° raster orientation experienced the highest increase of about 200% at a shear angle of 13.89°. This increase was related to severe localized plastic deformation, as seen in SEM imaging.

The results of this study, such as shear constitutive response, shear strength, shear zone, and shear strain at the end of shear zone, can be used for designing ABS parts that are subjected to shear loading in industrial applications. They can also be used in material models of the finite element analysis of fixtures and parts of real-life applications.

## Figures and Tables

**Table 1:** Printing parameters utilized in this study.

Printing Parameter	Value
Nozzle Temperature per ABS material	310 °C
Nozzle Temperature per Support material	300 °C
Printing Speed	168 mm/s
Layer Height	0.254 mm
Wall Thickness	0.914 mm
Bed Temperature	70 °C
Infill Pattern	Linear
Infill Type	Solid

**Table 2:** Dimensional parameters utilized for shear specimens.

Sample Dimensional Parameters (mm)	FHS-0	FHS-5.44	FHS-13.39	FHS-20.83
OD1	10	10.4	11.0	11.6
OD2	52	52	52	52
ID	10	10	10	10
L	32	32	32	32
B	13.9	13.9	13.9	13.9
H	24.2	24.2	24.2	24.2
A	6.00	6.00	6.00	6.00
RN	0.02	0.02	0.02	0.02
ND	0.04	0.04	0.04	0.04
Shzl	2.1	2.11	2.16	2.25
Shear Angle (°)	0	5.44	13.39	20.83
Shear Area (mm^2^)	25.2	25.32	25.92	27

**Table 3:** Shear zone length of all three specimen configurations.

	Shear Angle	Shear Zone Length (Radians)
Vertical	0°	0.14905
	5.44°	0.39695
	13.89°	0.45993
	20.83°	0.52151
+45/−45°	0°	0.1888
	5.44°	0.21505
	13.89°	0.68019
	20.83°	0.59524
0/90°	0°	0.36201
	5.44°	0.38002
	13.89°	0.58731
	20.83°	0.57262

## Data Availability

The datasets generated during and/or analyzed during the current study are available from the corresponding author on reasonable request.
