# Correction: Chalivendra et al. Effect of Shear Angle and Printing Orientation on Shear Constitutive Response of Additively Manufactured Acrylonitrile Butadiene Styrene. *Polymers* 2022, *14*, 2484

**DOI:** 10.3390/polym14153195

**Published:** 2022-08-05

**Authors:** Joshua Letizia, Vijaya Chalivendra, Dapeng Li

**Affiliations:** 1 Department of Mechanical Engineering, University of Massachusetts, Dartmouth, MA 02747, USA; 2 Department of Bioengineering, University of Massachusetts, Dartmouth, MA 02747, USA

In the original publication [1], there was a mistake in Figure 12 as published. Figure 8 was accidentally inserted instead of Figure 12 during the proofreading stage. We have modified the figure caption by deleting the following part “Zoomed in 0° filaments are in orange; 90° filaments are in red” because it is not necessary and adds confusion. Also, we added “of 0° filament” at the end of last line of the figure caption for clarity. The corrected Figure 12 and legend appears below. The authors apologize for any inconvenience caused and state that the scientific conclusions are unaffected. This correction was approved by the Academic Editor. The original publication has also been updated.

## Figures and Tables

**Figure 12:**
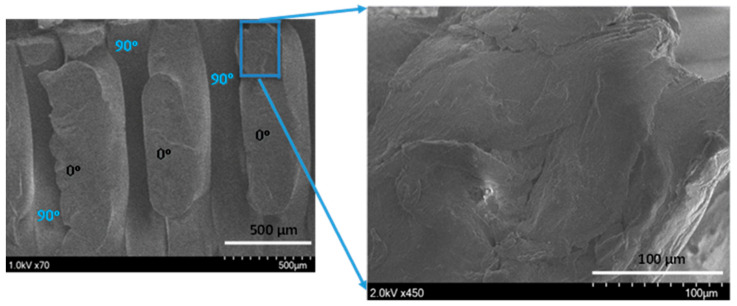
Scanning electron microscope snapshot of shear zone in the 0°/90° orientation with a 20.83° shear angle (**left**). Close up of shear zone of 0° filament (**right**).
